# Angiogenesis, cell differentiation and cell survival in tissue engineering and cancer research

**DOI:** 10.3205/iprs000067

**Published:** 2015-08-24

**Authors:** Daniel Johannes Tilkorn

**Affiliations:** 1 Klinik für Plastische, Rekonstruktive und Ästhetische Chirurgie, Handchirurgie, Alfried Krupp Krankenhaus, Essen, Germany

## Abstract

Recent medical advances lead to a growing demand for tissue engineering and regenerative medicine in the future. Tissue engineering and regenerative medicine aim to create substitute tissue or restore lost or impaired tissue by combining biological science with engineering techniques, whereas cancer research faces the challenge to identify and hinder aberrant and uncontrolled cell growth.

These two seemingly opposing fields of research share fundamental communalities.

This review focuses on the shared underlying biological processes. Exploring these mechanisms of tissue growth and homeostasis from different angles will allow for creative novel approaches for both areas of research.

## Zusammenfassung

Fortschritte in der Medizin lassen die Ansprüche an das Tissue Engineering und die Regenerative Medizin in Zukunft bedeutend steigen. Das Tissue Engineering und die Regenerative Medizin haben sich zur Aufgabe gestellt, Ersatzgewebe neu zu generieren, um erkranktes oder verloren gegangenes Gewebe wiederherzustellen, wohingegen die Tumorforschung die Herausforderung hat, Mechanismen zu identifizieren, die das unkontrollierte maligne Zellwachstum einschränken.

Diese beiden scheinbar gegensätzlichen Forschungszweige weisen fundamentale Gemeinsamkeiten bezüglich der zu Grunde liegenden biologischen Prozesse auf.

Ziel dieser Arbeit ist es, die biologischen Gemeinsamkeiten genauer zu untersuchen.

Die Betrachtung der Mechanismen des Gewebe-/Zellwachstums und der biologischen Homeostase aus unterschiedlichen Blickwinkeln kann zu kreativen, innovativen Forschungsansätzen in beiden Forschungsrichtungen führen.

## Introduction

The increase in life expectance and the ever growing medical progress allows an increased number of patients to survive cancer, severe trauma as well as chronic diseases, leading to a higher demand in substitute tissue to replace lost or diseased tissue or organs.

At present, these patients depend upon reconstructive procedures, requiring the transplantation of autologous or allogenic tissue. These procedures present some specific drawbacks such as donor site morbidity or adverse immunoreactions.

Tissue engineering and regenerative medicine combine engineering and biological principals, which aim to regenerate or create new tissue and organs using autologous cells to circumvent immunological reactions. In addition this tissue may also be used for diagnostic and research purposes.

It represents a great challenge for tissue engineering disciplines to create an ideal environment for cells to grow, survive and differentiate.

Stem or progenitor cells, extracellular matrices and a functional vascular network are essential prerequisites for successful generation of substitute tissue.

It is crucial for the development of clinically valuable tissue to enhance cell survival, differentiation as well as proliferation and to allow cell interaction, apoptosis and angiogenesis [1], [2], [3]. 

These biological processes mentioned above are oftentimes affected in tumor genesis, in which aberrant cell growth, unhindered cell proliferation, altered cell signaling and resistance towards apoptosis promote malignant growth [4].

In contrast to tissue engineering research the identification and inhibition of these underlying processes represent the greatest challenge for cancer research.

Even though tissue engineering and cancer research are seemingly opposing fields of research, there exist great similarity in regards to the underlying biological processes.

The unique properties of stem and progenitor cells and their role in tissue and organ regeneration bare great potential for engineering tissue constructs. On the other hand emerging evidence implicate stem cells and progenitor cells as the source of oncogenic transformation [5], [6], [7], [8], [9], [10]. A functional vascular network and successful neoangiogenesis is the essential basis for cell survival in vivo. Transplanted cells within tissue engineering constructs will not survive unless they are in close proximity or have access to a functional vascular network for nutritional and oxygen supply and waste deposition [11].

The same holds true for rapid growing malignant tumors.

The first vascular growth factors were discovered within well-vascularized tumors [12] and are now widely explored in the tissue engineering setting to enhance neoangiogenesis and cell survival in tissue constructs.

Cell signaling and cell differentiation play a significant and obvious role for cell based regenerative medicine [13], [14], [15] and have recently been introduced into the cancer setting.

Hence a profound knowledge of the fundamental biological process and interaction, which influence cell survival, differentiation and proliferation as well as apoptosis, is of great importance for both fields of research.

To further elucidate these fundamental communalities, a set of research experiments was conducted with a special focus on cell survival and differentiation both in tissue engineering constructs and tumor models.

The initial experiments assessed the role of angiogenesis, differentiation and cell survival in the tissue engineering setting. The gained knowledge was then translated into the cancer setting.

## Tissue engineering experiments

**Figure 1:**
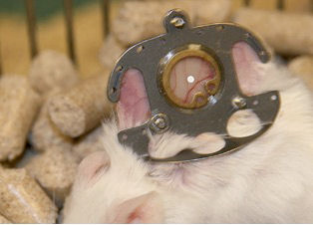
Dorsal skin fold chamber for intravital microscopy in situ

In order to evaluate the influence of various matrix materials on the angiogenic process, two different matrices, a lacto polymer and a PEGT/PBT copolymer were analyzed in an in vivo skin fold chamber in a mouse model. Intravital fluorescent microscopy allowed for a continuous analysis of the angiogenic process and the study of changes in microcirculation in vivo [16] (Figure 1 (Fig. 1)).

The angiogenic process was detected as early as on the first day with a peak on the seventh day when a functional vascular network has been formed.

**Figure 2:**
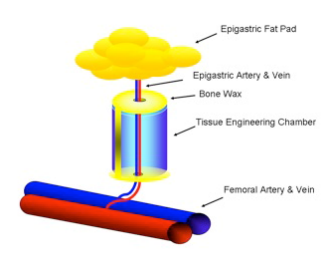
Tissue engineering chamber with intrinsic vascularisation centred around the superficial epigastic vessels

**Figure 3:**
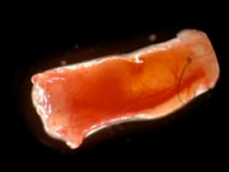
De novo generated tissue within the tissue engineering chamber model in mice

**Figure 4:**
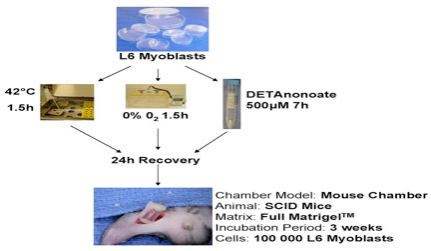
Experimental preconditioning regiment

**Figure 5:**
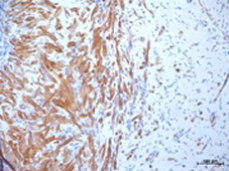
Differentiated myoblasts following hypoxic preconditioning and in vivo implantation

The importance of the vasculature on the survival of transplanted cells was verified for different cell types, myoblasts [17], thymocytes [18], osteoblasts and dental pulp cells [19], [20], using a tissue engineering model with intrinsic vascular supply. For this purpose the tissue engineering chamber was centered around superficial epigastric vessels as vascular pedicle in mice (Figure 2 (Fig. 2), Figure 3 (Fig. 3)). Further we could show the time relationship between angiogenesis and cell survival following cell implantation within a tissue engineering model in the groin of inbreed rats. An arteriovenous shunt was placed in a tissue engineering chamber on day 1 of the experiment. Myoblasts were inserted into the chamber on day 1, day 4 or day 7 according to experimental protocol. A marked increase in cell survival was detected when cells were implanted on day 4 or even more for cells implanted on the seventh day after establishing the tissue engineering construct, when a functional vascular network has been formed [17]. In order to detect cell innate factors able to improve cell survival under hypoxic conditions, myoblasts were preconditioned with either hypoxia, nitric oxide, hypoglycemia or hyperthermia prior to the implantation into the tissue engineering chamber (Figure 4 (Fig. 4)). Exposure to a sublethal dose of cell stress (preconditioning) significantly enhanced cell survival and led to a higher vascular density as well as cell differentiation [21] (Figure 5 (Fig. 5)).

In addition it was shown that adding dentin fragments as differentiation clues to the tissue engineering model apart from initiating cell differentiation, also positively influenced survival dental pulp stem cells [19], [20].

## Translation into cancer research

The knowledge gained from the tissue engineering experiments was transferred in the cancer setting to improve the reproducibility and comparability of sarcoma models.

An in vivo model with intrinsic vascular supply was developed in order to grow human sarcomas in mice and was compared to the commonly used subcutaneous implantation of tumor cells.

Tumors which had been grown in the model with intrinsic vascularization closely resembled the human primary tumor and allowed an improved evaluation of tumor angiogenesis and tumor matrix interaction [22], [23].

Finally tumor cells were shown to alter stromal cell differentiation in a chemokine fashion by adding cell free sarcoma media to preadipocytes in vitro [24].

## Conclusion

In conclusion a set of experiments demonstrated the importance of the biological process of angiogenesis, cell differentiation and cell survival both for tissue engineering and cancer research. Cellular pathways and mechanisms directing cell survival and function to successful tissue engineering outcomes are often examined in the cancer setting and vice versa. Acquiring insights in the biological processes to control the growth, differentiation and growth inhibition of a tissue engineered construct are challenges at the forefront of tissue engineering. Knowledge gained from cancer research have led to many novel insights including the identification of specific factors and signaling pathways that are aberrantly activated in the tumor. Elucidating these fundamental mechanisms of cancer development and tissue growth, homeostasis and regeneration will yield novel approaches for both areas of research. Many fundamental mechanisms of cancer development as well as tissue regeneration conjoin these opposing fields of research. Shining light on these mechanisms of tissue growth and homeostasis from different angles will allow for creative novel approaches for both areas of research [4].

## Notes

### Competing interests

The author confirms that there are no financial or non-financial conflicts of interest in regards to this article.
